# Translational Control of *Sox9* RNA by mTORC1 Contributes to Skeletogenesis

**DOI:** 10.1016/j.stemcr.2018.05.020

**Published:** 2018-06-28

**Authors:** Takashi Iezaki, Tetsuhiro Horie, Kazuya Fukasawa, Makoto Kitabatake, Yuka Nakamura, Gyujin Park, Yuki Onishi, Kakeru Ozaki, Takashi Kanayama, Manami Hiraiwa, Yuka Kitaguchi, Katsuyuki Kaneda, Takayuki Manabe, Yasuhito Ishigaki, Mutsuhito Ohno, Eiichi Hinoi

**Affiliations:** 1 Laboratory of Molecular Pharmacology, Division of Pharmaceutical Sciences, Kanazawa University Graduate School, Kakuma-machi, Kanazawa, Ishikawa 920-1192, Japan; 2 Venture Business Laboratory, Organization of Frontier Science and Innovation, Kanazawa University, Kanazawa, Ishikawa 920-1192, Japan; 3 Institute for Virus Research, Kyoto University, Kyoto 606-8507, Japan; 4 Medical Research Institute, Kanazawa Medical University, Kahoku, Ishikawa 920-0293, Japan; 5 Department of Neuroanatomy and Neuropharmacology, Faculty of Nursing, Chukyogakuin University, Mizunami, Gifu 509-6192, Japan

**Keywords:** mTORC1, translation, Sox9, undifferentiated mesenchymal cells

## Abstract

The mechanistic/mammalian target of rapamycin complex 1 (mTORC1) regulates cellular function in various cell types. Although the role of mTORC1 in skeletogenesis has been investigated previously, here we show a critical role of mTORC1/4E-BPs/SOX9 axis in regulating skeletogenesis through its expression in undifferentiated mesenchymal cells. Inactivation of *Raptor*, a component of mTORC1, in limb buds before mesenchymal condensations resulted in a marked loss of both cartilage and bone. Mechanistically, we demonstrated that mTORC1 selectively controls the RNA translation of *Sox9*, which harbors a 5′ terminal oligopyrimidine tract motif, via inhibition of the 4E-BPs. Indeed, introduction of *Sox9* or a knockdown of *4E-BP1/2* in undifferentiated mesenchymal cells markedly rescued the deficiency of the condensation observed in *Raptor*-deficient mice. Furthermore, introduction of the *Sox9* transgene rescued phenotypes of deficient skeletal growth in *Raptor*-deficient mice. These findings highlight a critical role of mTORC1 in mammalian skeletogenesis, at least in part, through translational control of *Sox9* RNA.

## Highlights

• mTORC1 controls skeletogenesis both in skeletogenic progenitors and in chondrocytes
• mTORC1/4E-BPs cascade regulates the translation of *Sox9* RNA
• SOX9 is a critical mediator in the control of skeletogenesis by mTORC1 *in vivo*

## Teaser

Iezaki et al. demonstrated that the mTORC1/SOX9 axis has essential roles in skeletal development through its expression in undifferentiated mesenchymal cells *in vivo*. Moreover, they identified that mTORC1/4E-BPs cascade regulates the translation of *Sox9* RNA in undifferentiated mesenchymal cells, highlighting a critical role of mTORC1/4E-BPs/SOX9 axis in regulating mammalian skeletogenesis.

## Introduction

The mechanistic/mammalian target of rapamycin (mTOR) is an evolutionally conserved serine/threonine kinase in eukaryotes, and it has been implicated in various physiological processes, such as growth, proliferation, survival, autophagy, and differentiation (Saxton and Sabatini, 2017). mTOR forms two separate complexes, designated mTOR complexes 1 and 2 (mTORC1 and mTORC2), which differ in their mechanisms of activation and downstream targets as well as in their composition (Brown et al., 1994, Sabatini et al., 1994). The RAPTOR subunit is specific to the mTORC1 complex, whereas the RICTOR subunit is specific to mTORC2 (Jacinto et al., 2004, Nojima et al., 2003). Tuberous sclerosis complex 1 (*Tsc1*) and complex 2 (*Tsc2*) are critical negative regulators of mTORC1 through their GTPase-activating protein activities toward the small G-protein Ras homolog enriched in the brain (Long et al., 2005). mTORC1 stimulates translation by phosphorylating downstream targets, including the eukaryotic translation initiation factor 4E (eIF4E)-binding proteins (4E-BPs) and the ribosomal protein S6 kinases (S6Ks), in response to various extracellular signals and intracellular cues. S6Ks phosphorylate components of the translational machinery and associated factors, such as ribosomal protein S6, eIF4B, and programmed cell death protein 4, leading to promotion of mRNA translation initiation (Dorrello et al., 2006, Holz et al., 2005). Phosphorylation of 4E-BPs by mTORC1 leads to their dissociation from eIF4E, thereby allowing association of eIF4E with eIF4G and assembly of the eIF4F translation initiation complex at the mRNA 5′ end (Brunn et al., 1997, Gingras et al., 1999).

Although global deletion of *Mtor* or *Raptor* results in embryonic lethality, cell-specific deletion strategies have revealed that mTORC1 is implicated in the pathogenesis of various diseases, including cancer, obesity, and cardiovascular disease, in addition to its physiological functions (Guertin et al., 2006, Murakami et al., 2004, Saxton and Sabatini, 2017). Genetic research recently revealed the critical role of mTORC1 in skeletogenesis through its expression in chondrocytes. This study showed that inactivation of mTORC1 signaling, by deleting either *Mtor* or *Raptor*, significantly diminished embryonic skeletal growth, causing delays in chondrocyte hypertrophy and bone formation (Chen and Long, 2014). Hyperactivation of mTORC1 by *Tsc1* deletion in chondrocytes led to chondrodysplasia with an uncoupling of the normal proliferation and differentiation program within the growth plate (Yan et al., 2016). Moreover, *Raptor* deficiency reduced the size of limb bud cells, resulting in overall diminution of the limb bud (Jiang et al., 2017). On the contrary, the precise underlying mechanisms of mTORC1 in skeletogenesis through its expression in skeletogenic progenitor cells (undifferentiated mesenchymal cells before chondrogenic mesenchymal condensation has occurred) remain unclear.

In our investigation of mTORC1 and its role in skeletogenesis, we found that the cartilage and bones of the appendicular skeleton were markedly diminished in *Raptor*-deficient mice. These mice were generated by a paired-related homeobox 1 (*Prx1*)-Cre-mediated recombination, which took place in undifferentiated limb bud mesenchymal cells before chondrogenic mesenchymal condensation occurred. Subsequent analyses determined that mTORC1 directly accelerates the translation of the sex-determining region Y-type high-mobility group box protein 9 (*Sox9*) RNA, which harbors the 5′ terminal oligopyrimidine tract (5′ TOP) motif in its 5′ UTR. This occurs through the phosphorylation of 4E-BPs, but not S6Ks in undifferentiated mesenchymal cells. Moreover, we uncovered that SOX9 is a critical mediator of mTORC1-dependent skeletogenesis and mesenchymal condensation *in vivo* and *in vitro*. Our results demonstrate that the translational control of *Sox9* is an essential pathway through which the mTORC1 pathway regulates skeletal development.

## Results

### Skeletal Development Requires the Expression of mTORC1 in Undifferentiated Mesenchymal Cells Prior to Chondrogenic Mesenchymal Condensation

To reveal the *in vivo* physiological role of mTOR in skeletal development, we inactivated the *Raptor* gene in undifferentiated limb mesenchymal cells or whole chondrocytes using *Prx1-Cre* transgenic mice (Logan et al., 2002) or collagen type II α1 (*Col2a1*)-*Cre* transgenic mice (Terpstra et al., 2003).

**Figure 1:**
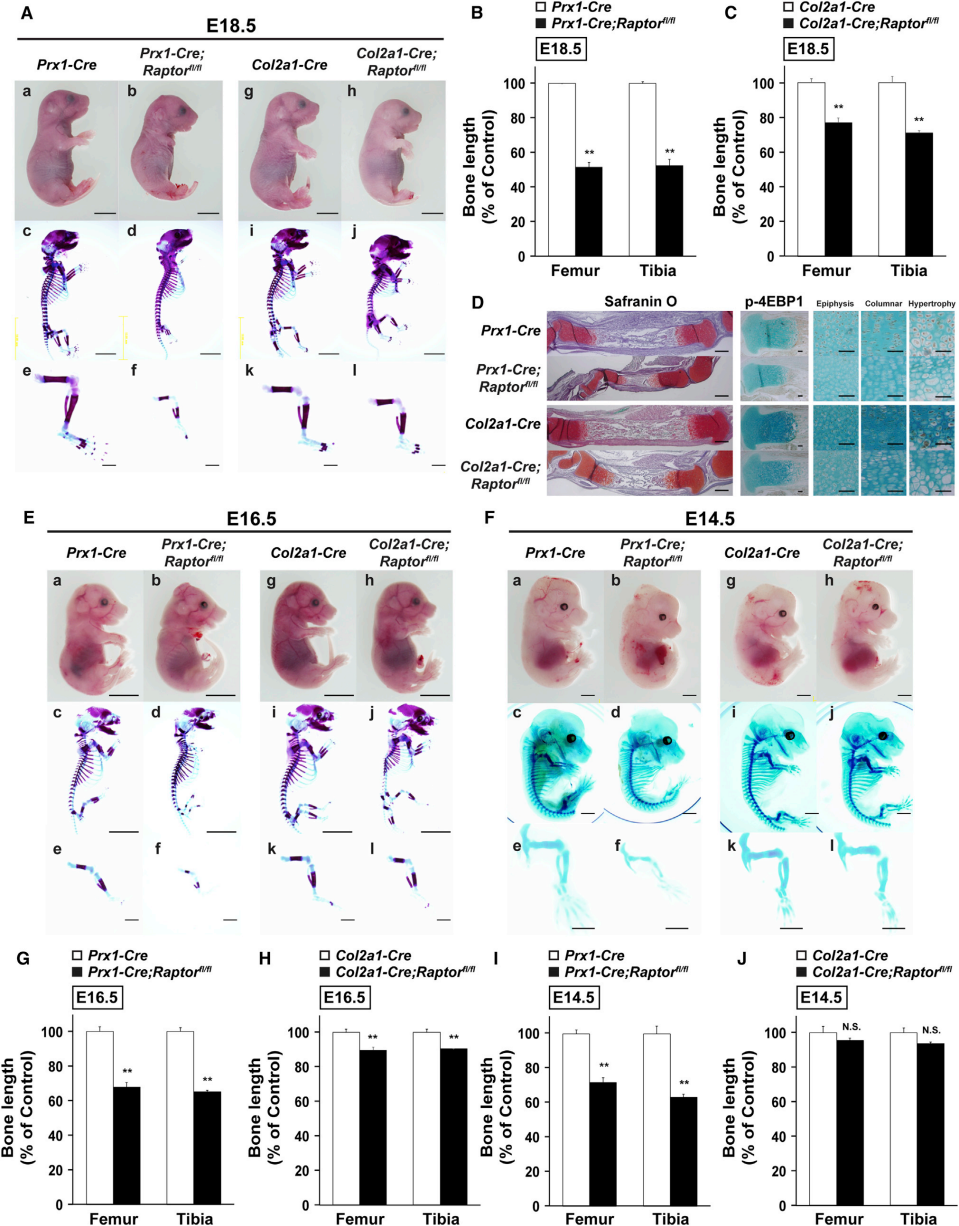
mTORC1 Is Essential for Skeletal Development before and after Mesenchymal Condensation (A–C) The whole and parts of skeleton of mutant embryos at E18.5. Embryos were double stained with alizarin red and Alcian blue (A). Scale bars, 5 mm (a and c) and 1 mm (e). Quantitative data of length of femur and tibia of (B) *Prx1-Cre;Raptor*^*fl/fl*^ embryos and (C) *Col2a1-Cre;Raptor*^*fl/fl*^ embryos at E18.5 (n = 4 independent samples). (D–J) Histological analyses of the tibia at E18.5 (D). Scale bars, 500 μm (safranin O) or 100 μm (p-4EBP1). The whole and parts of skeleton of mutant embryos at (E) E16.5 and (F) E14.5. Embryos were double stained with alizarin red and Alcian blue. (E) Scale bars, 5 mm (a and c) and 1 mm (e). (F) Scale bars, 1 mm (a, c, and e). Quantitative data of length of femur and tibia of mutant embryos at (G and H) E16.5 and (I and J) E14.5 (n = 4 independent samples). Representative images of skeletal preparations and histological analyses derived from more than four embryos from different litters are shown. ^∗∗^p < 0.01, significantly different from the value obtained in control embryos. Statistical significance was determined using the two-tailed, unpaired Student's t test. N.S., not significant.

*Prx1-Cre;Raptor*^*fl/fl*^ embryos at 18.5 days post-coitum (E18.5) had very short limbs and a deficiency of some of the craniofacial bones, but their appearance was otherwise normal. Staining of skeletal preparations from these embryos with Alcian blue and alizarin red indicated a marked shortage of all skeletal components in forelimbs and hindlimbs (Figures 1A and 1B), recapitulating previous findings that ablation of *Raptor* or *Mtor* in limb bud progenitors leads to short limbs (Chen and Long, 2014). On the contrary, *Co2a1-Cre;Raptor*^*fl/fl*^ embryos were characterized by a very severe and generalized chondrodysplasia. Alcian blue and alizarin red staining of skeletal preparations of E18.5 mutant embryos indicated that all the skeletal elements formed by endochondral ossification, including ribs, limbs, and vertebrae, were markedly smaller than embryos with typical growth (Figures 1A and 1C). In contrast, skeletal elements formed by intramembranous ossification lacked significant abnormalities. Histological analyses also revealed abnormalities in the long bones of both *Prx1-Cre;Raptor*^*fl/fl*^ embryos and *Co2a1-Cre;Raptor*^*fl/fl*^ embryos at E18.5 (Figures 1D and S1A). Phosphorylation status of 4E-BP1, a downstream effector of mTOR signaling, was markedly reduced in growth plate chondrocytes of *Raptor*-deficient embryos (*Prx1-Cre;Raptor*^*fl/fl*^ and *Co2a1-Cre;Raptor*^*fl/fl*^ embryos) at E18.5 (Figure 1D), indicating mTORC1 inactivation by *Raptor* deficiency. However, cell proliferation (PCNA-positive cells) of growth plate chondrocyte was comparable between wild-type (WT) and mutant embryos (Figure S1B), which is consistent with a previous study that showed normal cell proliferation of *Raptor*-deficient growth plate chondrocyte by bromodeoxyuridine-labeling assay (Chen and Long, 2014).

While defects in the formation of forelimbs and hindlimbs were already observed at E16.5 in both *Prx1-Cre;Raptor*^*fl/fl*^ and *Col2a1-Cre;Raptor*^*fl/fl*^ embryos (Figures 1E, 1G, and 1H), these types of defects were observed previously at E14.5 only in *Prx1-Cre;Raptor*^*fl/fl*^ embryos (Figures 1F and 1I). *Col2a1-Cre;Raptor*^*fl/fl*^ embryos were undistinguishable from WT embryos at E14.5 (Figures 1F and 1J).

Collectively, these results indicate that mTORC1 has essential roles in skeletal development both through its expression in undifferentiated mesenchymal cells prior to mesenchymal condensation, and through its expression in chondrocytes, as has been reported previously (Chen and Long, 2014, Jiang et al., 2017).

### mTORC1 Controls Mesenchymal Condensation and SOX9 Protein Levels in Mesenchymal Cells

**Figure 2:**
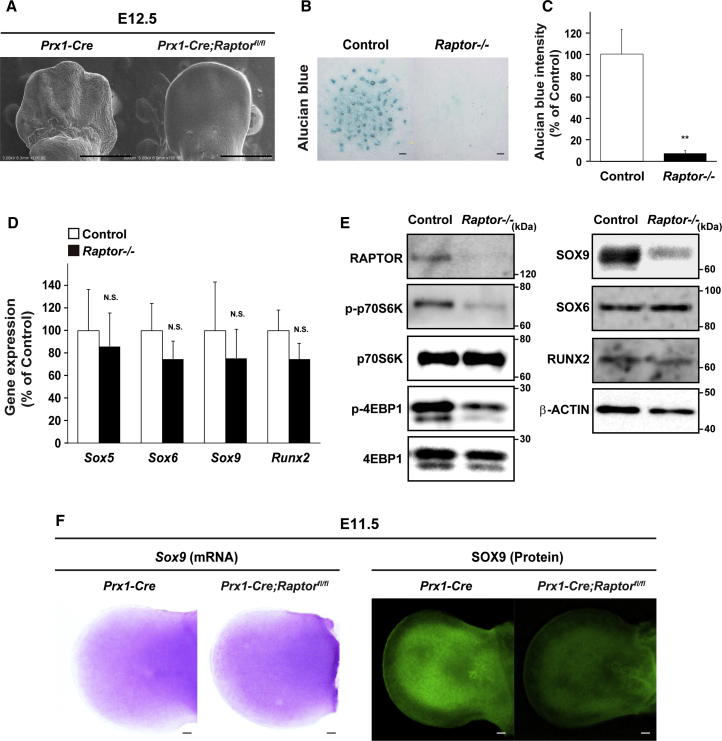
mTORC1 Controls Mesenchymal Condensation and SOX9 Protein Level in Mesenchymal Cells (A–E) Scanning electron microscope (SEM) analyses of forelimb buds of *Prx1-Cre;Raptor*^*fl/fl*^ embryos at E12.5 (A). Micromass culture of mesenchymal cells of *Prx1-Cre;Raptor*^*fl/fl*^ embryos at E12.5 was performed, followed by determination of (B and C) Alcian blue staining at day 6 (n = 4 independent experiments), (D) mRNA expression (n = 4 independent experiments), and (E) protein expression (n = 3 independent experiments). Scale bars, 100 μm. (F) Whole-mount *in situ* hybridization and immunohistochemical analyses of limb buds of *Prx1-Cre;Raptor*^*fl/fl*^ embryos at E11.5. Scale bars, 100 μm. ^∗∗^p < 0.01, significantly different from the value obtained in control cells. Statistical significance was determined using the two-tailed, unpaired Student's t test. N.S., not significant.

Further characterizing the precise role of mTORC1 expression in undifferentiated mesenchymal cells, scanning electron microscope (SEM) analysis showed that no distinct digit formation was observed in limb buds of *Prx1-Cre;Raptor*^*fl/fl*^ embryos at E12.5 (Figure 2A). Moreover, the numbers of Alcian blue-positive cells were decreased significantly by *Raptor* deficiency in an *in vitro* high-density micromass culture of limb bud cells prepared from E12.5 embryos (Figures 2B and 2C). These results indicate no discernible chondrogenic mesenchymal condensations in limb buds of *Prx1-Cre;Raptor*^*fl/fl*^ embryos at E12.5.

In *Raptor*-deficient mesenchymal cells, no significant changes were seen in the mRNA expressions of *Sox5, Sox6, Sox9*, or *Runx2*, pivotal transcription factors for skeletogenesis (Akiyama et al., 2002, Smits et al., 2001, Takarada et al., 2016), compared with expressions in WT cells (Figure 2D). In contrast, SOX9 protein expression was markedly decreased in *Raptor*-deficient cells, despite the lack of change in RUNX2 and SOX6 protein expression (Figure 2E), in accordance with the repression of the downstream phosphorylation of mTORC1, p70S6K, and 4E-BP1 by *Raptor* deficiency. Moreover, *Sox9* mRNA expression in limb buds was comparable between WT and *Prx1-Cre;Raptor*^*fl/fl*^ embryos at E11.5, which is inconsistent with a previous study that showed that *Sox9* mRNA expression domain appears to be reduced in the limb bud of *Raptor*-deficient embryos at E11.75 (Jiang et al., 2017). Contrary to *Sox9* mRNA expression, SOX9 protein expression was significantly decreased in mutant embryos compared with WT embryos (Figure 2F). Moreover, the level of COL2, the transcriptional target of SOX9, also decreased in mutant limb buds (Figure S1C).

These results indicate that mTORC1 markedly controls mesenchymal condensation and preferentially regulates SOX9 protein levels, but not mRNA levels, through its expression in undifferentiated mesenchymal cells *in vitro* and *in vivo*.

### The Pathway for 4E-BPs Is Implicated in the mTORC1-Dependent Control of SOX9 Protein Levels and Mesenchymal Condensation

mTORC1 is well known for controlling the translation of specific mRNAs by phosphorylating downstream substrates, including S6Ks and 4E-BPs. S6K1 phosphorylates and activates several substrates that promote mRNA translation initiation, including eIF4B, which positively regulates the 5′ cap binding eIF4F complex, while 4E-BPs inhibit translation by binding and sequestering eIF4E to prevent assembly of the eIF4F complex (Gingras et al., 1999, Holz et al., 2005).

**Figure 3:**
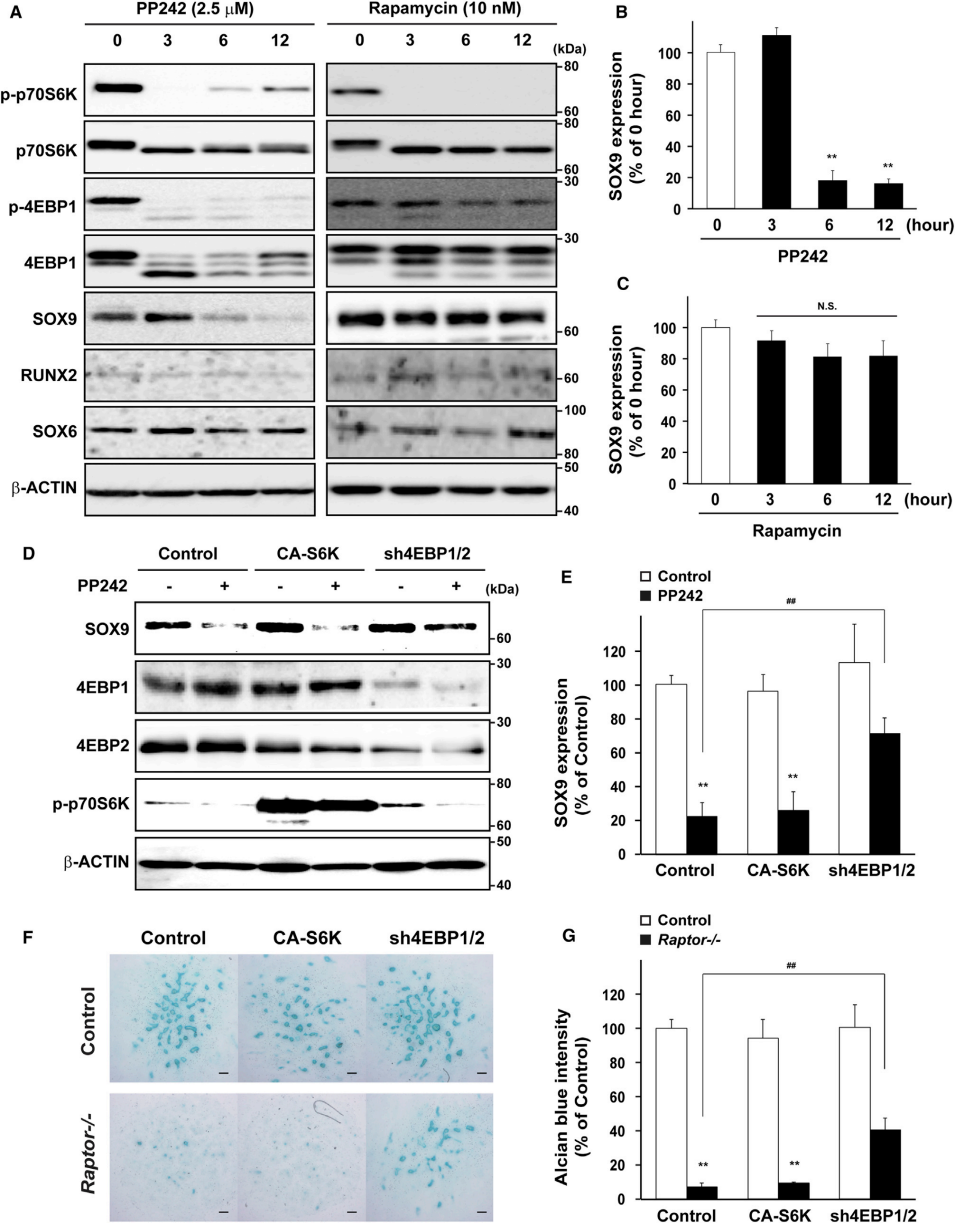
4E-BPs Pathway Is Implicated in the mTORC1-Dependent Control of SOX9 Protein Level and Mesenchymal Condensation (A–C) Primary mesenchymal cells from WT embryos were treated with PP242 at 2.5 μM or rapamycin at 10 nM for the indicated hours, and subsequent immunoblotting (n = 4 independent experiments). (D and E) Primary mesenchymal cells were retrovirally transfected with *CA-S6K* or *sh4EBP1/2*, and subsequently treated with PP242, followed by immunoblotting (n = 4 independent experiments). (F and G) Primary mesenchymal cells were retrovirally infected with *CA-S6K* or *sh4EBP1/2*, and subsequent micromass culture, followed by Alcian blue staining (n = 4 independent experiments). Scale bars, 100 μm. ^∗∗^p < 0.01, significantly different from the value obtained in control cells. ^##^p < 0.01, significantly different from the value obtained in (E) PP242-treated cells transfected with empty vector or (G) *Raptor*-deficient cells transfected with empty vector. Statistical significance was determined using the one-way ANOVA with the Bonferroni *post-hoc* test. N.S., not significant.

To investigate whether mTORC1 specifically regulates the translation of *Sox9* mRNA, mesenchymal cells were treated with PP242, an active-site mTOR inhibitor, or rapamycin, an allosteric mTOR inhibitor, followed by subsequent examination of protein expression. Treatment with PP242 decreased the phosphorylation of both 4E-BP1 and p70S6K, while rapamycin selectively repressed the phosphorylation of p70S6K. Under these experimental conditions, SOX9 protein level was repressed by PP242, but not by rapamycin, despite no changes in protein levels for RUNX2 and SOX6 (Figures 3A–3C).

Next, we determined genetically which pathways are associated with the regulation of SOX9 protein levels by mTORC1. To that end, mesenchymal cells were retrovirally infected with the constitutive active form of S6K (*CA-S6K*) or *sh4E-BP1/2*, and then subsequently treated with PP242, followed by determination of SOX9 expression. PP242 treatment decreased SOX9 protein levels in CA-S6K-infected cells, as was observed in control cells, but a double knockdown of *4E-BP1* and *4E-BP2*, which have functional redundancy, significantly prevented the PP242-induced repression of SOX9 protein levels (Figures 3D and 3E).

In accordance with SOX9 expression, the repression of mesenchymal condensation was significantly prevented by the double knockdown of *4E-BP1* and *4E-BP2*, but not by *CA-S6K* in *Raptor*-deficient cells (Figures 3F and 3G).

Altogether, these pharmacological and genetic analyses indicated that mTORC1-dependent control of *Sox9* mRNA translation and mesenchymal condensation could be primarily mediated through the 4E-BPs pathway, but not the S6K pathway.

### Translation of *Sox9* RNA Is Controlled by mTORC1

**Figure 4:**
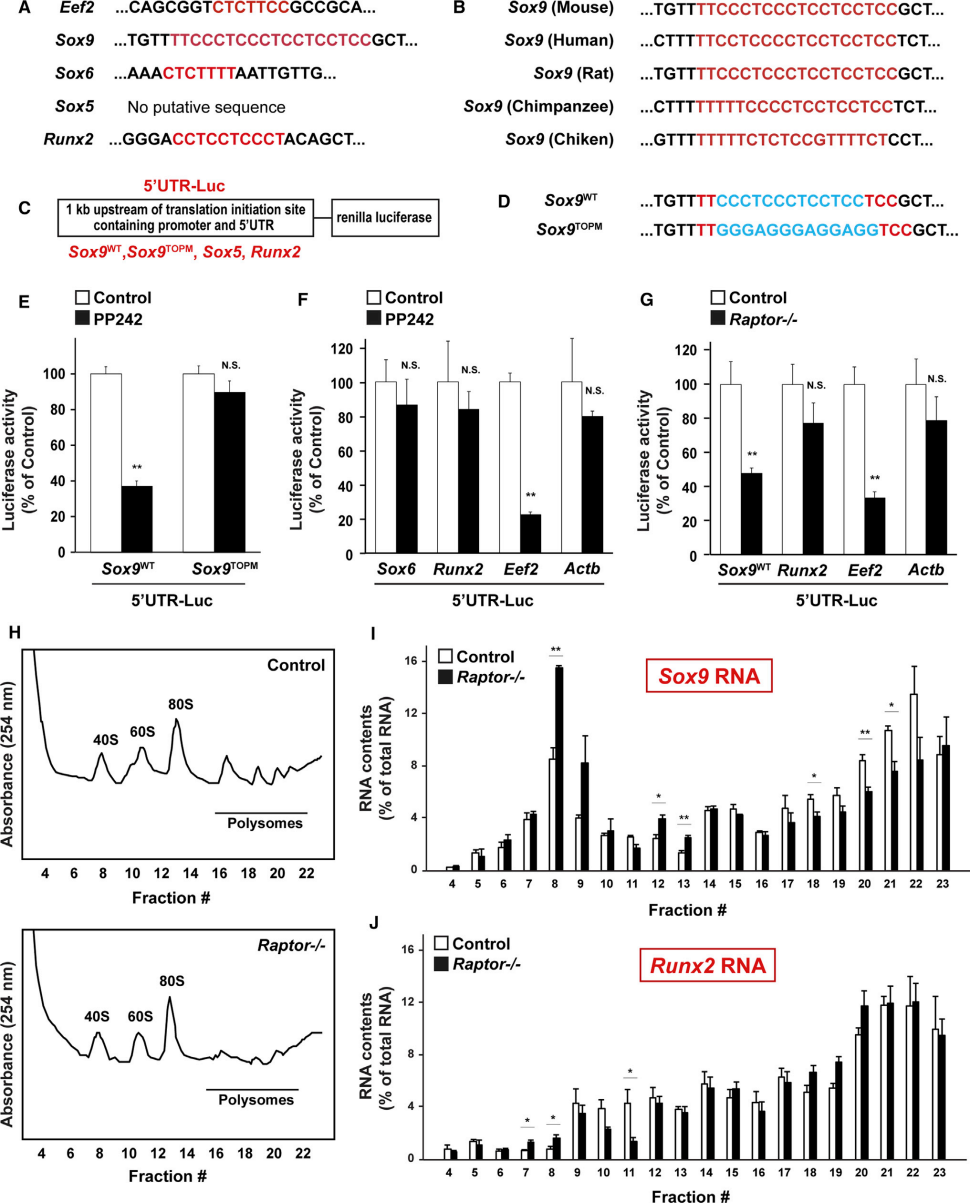
*Sox9* RNA Is Translationally Controlled by mTORC1 (A) 5′ TOP or TOP-like motifs in each gene. (B) Conservation of 5′ TOP or TOP-like motif in *Sox9* among vertebrates. (C) Reporter constructs containing the promoter and 5′ UTR of each gene followed by an open reading frame encoding renilla luciferase. (D) WT and mutant 5′ TOP motif of *Sox9* (Sox9^WT^ and Sox9^TOPM^). (E and F) WT mesenchymal cells were transfected with the indicated reporter constructs, and then treated with PP242, followed by analysis for luciferase activity (n = 4 independent experiments). (G) Mesenchymal cells of *Prx1-Cre;Raptor*^*fl/fl*^ embryos at E12.5 were transfected with the indicated reporter constructs, followed by analysis for luciferase activity (n = 4 independent experiments). (H–J) Polysomes from WT cells and *Raptor*-deficient cells were sedimented in sucrose density gradients to separate efficiently 40S, 60S, 80S, and polysomes, followed by determination of distribution of RNA contents (n = 3 independent experiments). ^∗^p < 0.05, ^∗∗^p < 0.01, significantly different from the value obtained in control cells. Statistical significance was determined using the two-tailed, unpaired Student's t test. N.S., not significant.

It has been suggested recently that mTORC1 translationally controls the clear majority of mRNAs possessing a 5′ TOP and/or a pyrimidine-rich translational element (Hsieh et al., 2012, Thoreen et al., 2012). TOP mRNAs are defined as those with a cytidine immediately after the 5′ cap, followed by an uninterrupted stretch of 4–14 pyrimidine nucleotides, and they tend to encode proteins associated with translation (Hay and Sonenberg, 2004, Meyuhas, 2000). We computationally analyzed 5′ flanking regions of genes related to skeletogenesis, and then identified 5′ TOP or TOP-like motifs in the 5′ flanking region of *Sox9*, *Sox6*, and *Runx2*. This has been seen in eukaryotic elongation factor 2 (*Eef2*), an established TOP mRNA, but not in *Sox5* (Figure 4A). In the *Sox9* 5′ flanking region, 5′ TOP or TOP-like motifs were conserved in different species (Figure 4B). To reveal whether 5′ TOP or TOP-like motifs of *Sox9*, *Sox6*, and *Runx2* contribute to the mTORC1-dependent translational control of these mRNAs, reporter vectors were constructed by cloning the 1-kb fragment upstream of the translation initiation site containing the promoter and the 5′ UTR of each gene into the upstream section of the open reading frame encoding *Renilla* luciferase (designated as 5′ UTR-Luc) (Figure 4C). The PP242 treatment abolished reporter activities of *Eef2* and *Sox9*, but not of *Runx2* and *Actb* (Figures 4E and 4F). On the contrary, PP242 did not alter the reporter activity of *Sox9* with the mutation in the 5′ TOP sequence (*Sox9*^TOPM^) (Figures 4D and 4E). Furthermore, *Raptor* deficiency impaired the reporter activities of *Eef2* and *Sox9*, but not of *Runx2* and *Actb* (Figure 4G).

To directly investigate if mTORC1 controls the translation of *Sox9* mRNA, polysomes from WT cells and *Raptor*-deficient cells were sedimented in sucrose density gradients to efficiently separate 40S, 60S, 80S, and polysomes, followed by a determination of the distribution of RNA contents. As shown in Figure 4H, *Raptor*-deficient cells showed a decrease in polysome content compared with WT cells. Next, we assessed the effects of *Raptor* deficiency on the distribution of RNA contents in *Sox9*, *Runx2*, *Sox5*, *Sox6*, *Actb*, and *Eef2*. *Raptor* deficiency caused a significant decrease in *Sox9* RNA contents in polysome fractions, but led to an increase in the 40S-80S fractions (Figure 4I), corresponding to the alteration of RNA distribution in *Eef2* (Figure S2D). Showing consistency with the results of no changes in 5′ UTR-Luc activities in *Runx2* and *Sox6*, and no putative 5′ TOP or TOP-like motifs in *Sox5* (Figures 4A, 4F, and 4G), *Raptor* deficiency failed to shift the distribution of RNA contents in *Runx2* (Figure 4J), *Sox5*, *Sox6*, in addition to *Actb* mRNA (Figures S2A–S2C). These results indicate that mTORC1 preferentially controls the translation of *Sox9* mRNA, but not of *Runx2*, *Sox6*, and *Sox5* mRNA.

### SOX9 Contributes to mTORC1-Dependent Mesenchymal Condensation and Skeletal Development

**Figure 5:**
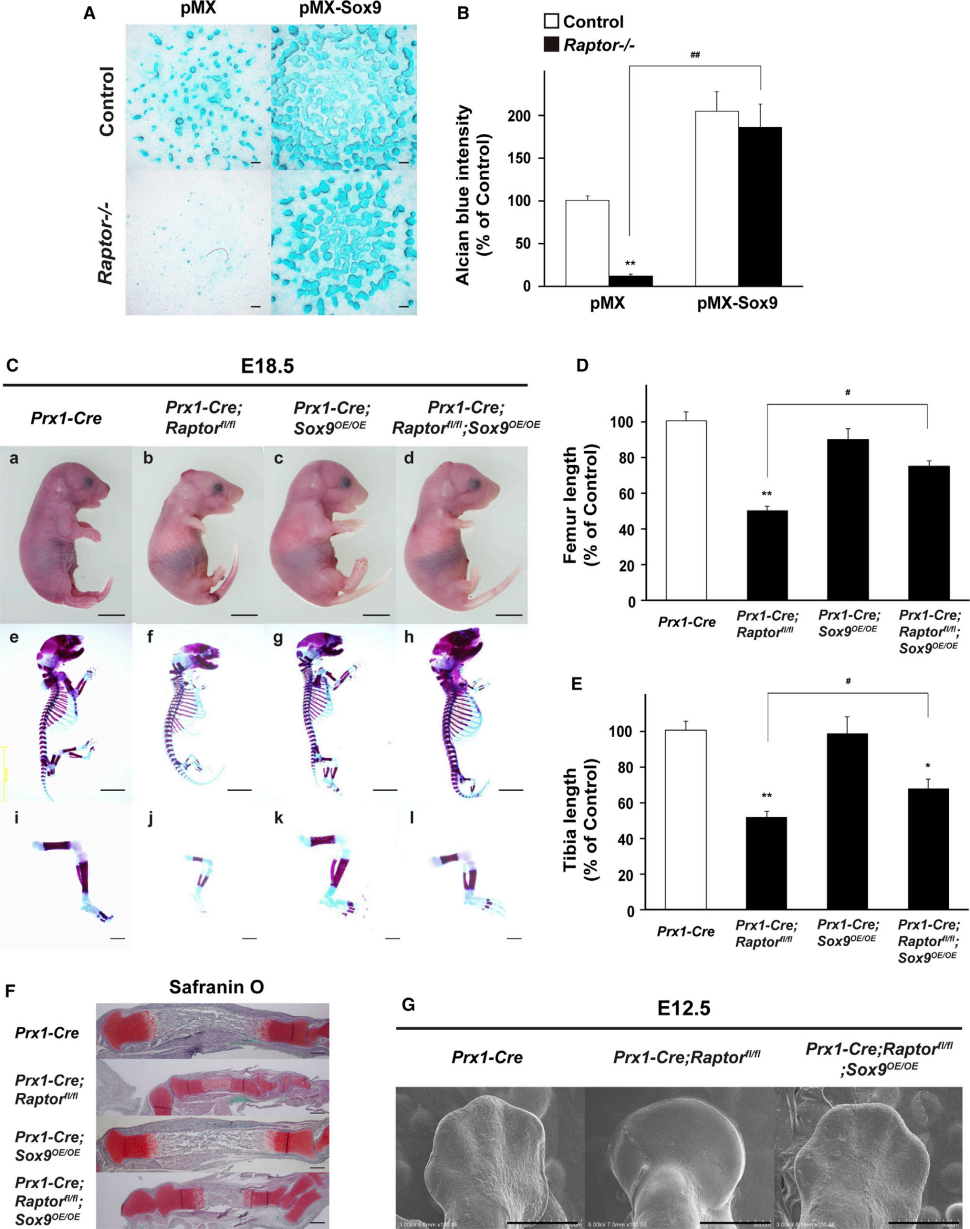
SOX9 Contributes to mTORC1-Dependent Mesenchymal Condensation and Skeletal Development (A and B) Mesenchymal cells from *Prx1-Cre;Raptor*^*fl/fl*^ embryos were retrovirally infected with *Sox9* expression vector, and subsequent micromass culture, followed by Alcian blue staining at day 6 (n = 4 independent experiments). Scale bars, 100 μm. (C–E) The whole and parts of skeleton of mutant embryos at E18.5. Embryos were double stained with alizarin red and Alcian blue. Scale bars, 10 mm (a and e) and 1 mm (i). Quantitative data of length of (D) femur and (E) tibia of mutant embryos at E18.5 (n = 4 independent samples). (F) Histological analyses of the tibia at E18.5. Scale bars, 500 μm. Representative images of skeletal preparations and histological analyses derived from more than four embryos from different litters are shown. (G) SEM analyses of forelimb buds of mutant embryos at E12.5. Scale bars, 500 μm. (B) ^∗∗^p < 0.01, significantly different from the value obtained in control cells. ^##^p < 0.01, significantly different from the value obtained in *Raptor*-deficient cells transfected with empty vector. (D and E) ^∗^p < 0.05, ^∗∗^p < 0.01, significantly different from the value obtained in control embryos. ^#^p < 0.05, significantly different from the value obtained in *Prx1-Cre;Raptor*^*fl/fl*^ embryos. Statistical significance was determined using the one-way ANOVA with the Bonferroni *post-hoc* test.

Given that SOX9 is essential for mesenchymal condensation and skeletal development (Akiyama et al., 2002), and that translational control of *Sox9* mRNA occurs through mTORC1 (Figure 4), we next determined whether the control of *Sox9* translation underlines the effects of the mTORC1 pathway on mesenchymal condensation and skeletal development. To that end, we used a *Sox9* cDNA lacking the 5′ UTR that harbors 5′ TOP elements. Consistent with previous findings, *Sox9* overexpression increased the intensity of Alcian blue in WT cells, but, more importantly, *Sox9* overexpression significantly attenuated the repression of mesenchymal condensation by *Raptor* deficiency (Figures 5A and 5B).

Finally, we examined whether this is genetically viable *in vivo*. To that end, the Cre-inducible *Sox9* transgene with the human cytomegalovirus enhancer (hereafter referred to as *Sox9*^*OE*^), which lacks the 5′ TOP elements of *Sox9*, was introduced into *Prx1-Cre;Raptor*^*fl/fl*^ embryos. As is evident in Figures 5C–5F, axial skeletal abnormalities in *Prx1-Cre;Raptor*^*fl/fl*^ embryos were markedly rescued by the forced conditional expression of *Sox9* (*Prx1-Cre;Raptor*^*fl/fl*^;*Sox9*^*OE/OE*^ embryos) at E18.5. Moreover, SEM analysis of limb buds at E12.5 showed a distinct digit formation in the limbs of *Prx1-Cre;Raptor*^*fl/fl*^;*Sox9*^*OE/OE*^ embryos, as was seen in control embryos (Figure 5G), but no distinct digit formation was seen in the limb buds of *Prx1-Cre;Raptor*^*fl/fl*^ embryos.

Taken together, these data provide genetic evidence that SOX9 can contribute to mTORC1-dependent mesenchymal condensation and skeletal development *in vitro* and *in vivo.*

## Discussion

Although previous genetic and pharmacological studies have revealed the crucial role of mTORC1 in skeletal development (Chen and Long, 2014, Jiang et al., 2017, Phornphutkul et al., 2008, Phornphutkul et al., 2009, Yan et al., 2016), here we provide evidence demonstrating the critical role of the mTORC1/4E-BPs/SOX9 axis in skeletogenesis. Although it is well-established that *Sox9*/SOX9 is the subject of many types of regulatory mechanisms, including epigenetic and transcriptional regulation of its gene, post-transcriptional regulation of its RNA, and post-translational modifications of its protein (Akiyama and Lefebvre, 2011, Kozhemyakina et al., 2015, Lefebvre and Dvir-Ginzberg, 2017), no previous literature has reported the translational control of *Sox9* RNA thus far. Herein, using a battery of well-established biochemical and functional assays, several lines of evidence indicate that the mTORC1/4E-BPs cascade regulates the translation of *Sox9* RNA in undifferentiated mesenchymal cells. First, *Raptor* deficiency markedly abolished the SOX9 protein, but not *Sox9* mRNA, in both mesenchymal cells and limb buds (Figure 2). Second, an active-site mTOR inhibitor, PP242, suppressed the reporter activity of 5′ UTR-Sox9-luc, which harbors its promoter and the 5′ UTR, including the 5′ TOP motif (Figure 4). Third, and most importantly, *Raptor* deficiency abolished the *Sox9* RNA contents in polysome fractions (Figure 4). However, given that mTORC1 controls protein catabolism, most notably by autophagy, and protein turnover by a ubiquitin-proteasome system (Kim et al., 2011a, Martina et al., 2012, Zhao et al., 2015), the possibility that *Raptor* deficiency may affect catabolism and/or turnover of the Sox9 protein in mesenchymal cells should not be excluded.

*Prx1-Cre;Raptor*^*fl/fl*^ embryos showed a marked loss of all skeletal components in both fore- and hindlimbs (Figure 1), resembling the mutant mice that developed from the inactivation of *Sox9* in undifferentiated limb bud mesenchymal cells (*Prx1-Cre;Sox9*^*fl/fl*^ embryos) (Akiyama et al., 2002). However, the phenotype of the *Raptor*-deficient embryos was milder than that caused by the *Sox9* deficiency in terms of axial skeletal abnormalities. In addition, *in vivo* introduction of *Sox9* did not completely rescue the loss of skeletal components in *Prx1-Cre;Raptor*^*fl/fl*^ embryos. We, therefore, cannot exclude the possibility that another pathway also may contribute to the regulation of mTORC1-dependent skeletogenesis. Indeed, it has been reported that mTORC1 controls bone development through the S6K1/RUNX2 axis in pre-osteoblasts (Dai et al., 2017) or the S6K1/Gli2 axis in chondrocytes (Yan et al., 2016), despite no significant changes in the expression of *Runx2* in *Raptor*-deficient mesenchymal cells (Figure 2), and no significant rescue of mesenchymal condensation by introducing *CA-S6K* into *Raptor*-deficient cells (Figure 3) in this study.

Rapamycin, an allosteric mTOR inhibitor, potently prevents the phosphorylation of S6K1, while it has modest or little inhibitory effect on the phosphorylation of 4E-BP1. On the contrary, PP242, an active-site mTOR inhibitor, inhibits the phosphorylation of both S6K1 and 4E-BP1, indicating that PP242 could impair the translation of RNA to a much greater degree than could rapamycin, largely owing to their inhibition of rapamycin-resistant functions of mTORC1 (Choo et al., 2008, Thoreen et al., 2009).

It has previously been shown that discrete *Sox9* mRNA expression domain demarcating digit primordia do not appear in *Raptor*-deficient embryos at E11.75 (Jiang et al., 2017), whereas we demonstrated that *Sox9* mRNA expression in limb buds was comparable between WT and *Prx1-Cre;Raptor*^*fl/fl*^ embryos at E11.5 (Figure 2). The discrepancy may be probably explained by the different embryonic stages analyzed.

In conclusion, the mTORC1/4E-BPs pathway is critical for skeletogenesis *in vivo* by modulating the translation of *Sox9* mRNA through its expression in undifferentiated mesenchymal cells. Although further studies should be performed to determine how mTORC1 activity is regulated in undifferentiated mesenchymal cells, BMP and FGF signals could be candidates among multiple signals to modulate mTORC1 activity, in addition to hormonal and nutrient signals (Lin et al., 2011, Wang et al., 2017). Our findings may contribute to an improved understanding of the molecular mechanisms underlying skeletal development, as well as opportunities for the development of drugs targeting cartilage diseases associated with the abnormal expression or functioning of SOX9 in humans, such as campomelic dysplasia (Wagner et al., 1994). Moreover, SOX9 as well as mTORC1, is associated with tumorigenesis in several cancers (Grabiner et al., 2014, Jo et al., 2014, Pritchett et al., 2011, Sato et al., 2010), identifying the mTORC1/4E-BPs/SOX9 cascade as a prominent candidate for developing drugs targeting various human diseases.

## Experimental Procedures

### Mice

*Raptor*^*fl/fl*^, and *Sox9*^*OE/OE*^ mice were crossed with either *Prx1-Cre* or *Col2a1-Cre* mice (Kim et al., 2011b, Logan et al., 2002, Terpstra et al., 2003). These mutant mice were backcrossed for more than five generations with C57BL/6J. Mice were bred under standard animal housing conditions at 23°C ± 1°C with humidity of 55% and a light/dark cycle of 12 hr, with free access to food and water. Genotyping was performed by PCR using tail genomic DNA. The study protocol meets the guidelines of the Japanese Pharmacological Society and was approved by the Committee for Ethical Use of Experimental Animals at Kanazawa University, Kanazawa, Japan. The numbers of animals used per experiment are stated in the figure legends.

### Skeletal Preparation and Histological Analyses

Embryos were eviscerated and the skins were removed, and subsequent fixation in 95% ethanol overnight. Embryos were then immersed in Alcian blue solution overnight, and were transferred in a solution of 2% KOH, followed by staining in alizarin red solution overnight. Finally, the skeletons were cleaned with 1% KOH/20% glycerol and then stored in 50% ethanol/50% glycerol (Iezaki et al., 2016).

Mouse tibiae were fixed with 10% formalin neutral buffer solution, embedded in paraffin, and then sectioned at a thickness of 5  μm. Sections were stained with safranin O and von Kossa (Wang et al., 2005). Sections were incubated in a blocking buffer for 1 hr, followed by incubation with anti-phospho-4EBP1 antibody (no. 2855) (Cell Signaling Technologies) or anti-PCNA antibody (no. ab18197) (Abcam) for 24 hr. After washing with PBS, sections were further treated for 2 hr with biotin-conjugated anti-rabbit immunoglobulin G (IgG) (no. BA-1000) (Vector), and subsequent incubation with streptavidin-horseradish peroxidase.

### Generation of Retroviral Vectors and Infection

pMX-CA-S6K and pMX-Sox9 vectors were generated by subcloning into pMX vector from pRK7-HA-S6K1-F5A-E389-R3A (no. 8991) and pWPXL-Sox9 (no. 36979) vectors, respectively, which were obtained from Addgene. The oligonucleotides for 4E-BP1 short hairpin RNA (shRNA) and 4E-BP2 shRNA were synthesized (Table S1), annealed, and inserted into the RNAi-Ready pSIREN-RetroQ vector. These vectors were then transfected into PLAT-E cells using the calcium carbonate method. Virus supernatant was collected 48 hr after transfection and cells were then infected with this viral supernatant in the presence of 4 μg/mL polybrene (Fukasawa et al., 2016).

### Micromass Culture System

Limb bud mesenchymal cells from E12.5 were treated with 0.1% collagenase and 0.1% dispase for 1.5 hr at 37°C, and subsequently resuspended in DMEM/F12 medium containing 10% fetal bovine serum (FBS) at 1.5 × 10^7^ cells/mL. Droplets were carefully placed in each well interior of a 4-well plate. Cells were allowed to adhere at 37°C for 1.5 hr, followed by the addition of chondrogenic medium (DMEM/F12 medium containing 10% FBS and 50 μg/mL ascorbic acid). Medium was changed every 2 days, and Alcian blue stain was performed on day 6.

### Real-Time qPCR

Total RNA was extracted from cells, followed by the synthesis of cDNA using reverse transcriptase and oligo-dT primer. The cDNA samples were then used as templates for real-time PCR analysis, which was performed on an MX3005P qPCR System (Agilent Technologies) using specific primers for each gene (Table S2). Expression levels of the genes examined were normalized using *Actb* expression as an internal control for each sample.

### Immunoblotting Analysis

Cells were solubilized in lysis buffer containing 1% Nonidet P-40. Samples were then subjected to SDS-PAGE, followed by transfer to polyvinylidene difluoride membranes and subsequent immunoblotting. The primary antibodies used were, anti-RUNX2 (no. 12556), anti-RAPTOR (no. 2280), anti-phospho-p70S6K1 (no. 9205), anti-p70S6K1 (no. 2708), anti-phospho-4EBP1 (no. 2855), anti-4EBP1 (no. 9644), and anti-4EBP2 (no. 2845) (Cell Signaling Technologies), anti-β-actin (no. sc-47778) and anti-SOX6 (no. sc-17332) (Santa Cruz Biotechnology), anti-SOX9 (no. AB5535) (EMD Millipore).

### Luciferase Assay

Plasmids pIS1 (no. 12179), pIS1-Eef25UTR-renilla (no. 38235), and pIS1-Actb5UTR-renilla (no. 38234) were obtained from Addgene, and plasmids 5′ UTR-Runx2-luc, 5′ UTR-Sox6-luc and 5′ UTR-Sox9-luc were generated by PCR-based cloning with mouse genomic DNA. To generate 5′ UTR-Sox9^TOPM^-luc, site-directed mutagenesis was performed using the PrimeSTAR Max Mutagenesis Basal Kit (Takara Bio) with specific primers (Table S3). For the luciferase assay, cells were transfected with reporter vectors using the lipofection method as previously described (Hinoi et al., 2014), followed by the preparation of cell lysates and subsequent determination of luciferase activity using specific substrates in a luminometer (ATTO). Transfection efficiency was normalized by determining the LacZ activity.

### Polysome Fraction

Cells were incubated with 100 μg/mL cycloheximide for 5 min at 37°C, and subsequently solubilized in lysis buffer (20 mM HEPES-NaOH [pH 7.5], 100 mM KCl, 10 mM MgCl_2_, 1 mM DTT, 0.25% NP-40, 100 μg/mL cycloheximide) containing protease inhibitor cocktail and RNase inhibitor. The lysate was clarified by centrifugation at 14,000 rpm for 10 min, and the supernatant was then loaded onto ultracentrifuge tubes containing sucrose gradient buffer (10%–40% sucrose) and centrifuged at 40,000 rpm for 2.5 hr with an SW41 rotor. Fraction samples were collected and RNA was extracted with TRI Reagent LS (Sigma). The RNA samples were used for real-time PCR analysis using KAPA SYBR Universal One-Step RT-qPCR Kit (KAPA Biosystems) with specific primers for each gene (Table S4).

### SEM Observation with Ionic Liquid

Ionic liquid (IL), HILEM IL1000 (Hitachi High-Technologies) was used as coating reagent as described in previous papers (Ishigaki et al., 2011, Tsuda et al., 2011). In brief, this IL was mixed with pure water prior to use. The mixture was then pre-warmed at 40°C in a block heater to decrease its viscosity. This IL-water mixture was dropped onto the fixed samples and left for 1 min at room temperature. Excess IL was absorbed by paper and blowing away by a hairdryer. Then, the sample was evenly pasted onto an electrical conductive tape (Nisshin EM) that was put on an aluminum sample stub for SEM. A Hitachi S3400N SEM (Hitachi High-Technologies) was used in this study.

### Whole-Mount *In Situ* Hybridization and Immunohistochemistry

For whole-mount *in situ* hybridization, limb buds were fixed with 4% paraformaldehyde, dehydrated in methanol, rehydrated by a series of methanol/PBS gradient, and treated with 0.2 M HCl and 10 μg/mL proteinase K. Limb buds were then subjected to acetylation in 0.1 M triethanolamine with 0.25% acetic anhydride. After prehybridization, limb buds were incubated with digoxigenin-labeled cRNA probe at 60°C for 16 hr and subsequently treated with 4 μg/mL RNase A. Limb buds were further incubated with anti-digoxigenin-ALP Fab fragments for 16 hr, followed by treatment with NBT/BCIP.

For whole-mount immunohistochemistry, limb buds were fixed with methanol:DMSO (4:1) overnight, and washed three times with 1% Triton X-100/PBS. After treatment with blocking buffer (PBS containing 1% Triton X-100 and 5% normal goat serum) for 1 hr, limb buds were incubated with anti-SOX9 antibody (no. AB5535) (EMD Millipore) or anti-COL2 antibody (no. ab34712) (Abcam) in blocking buffer for 24 hr. Limb buds were further incubated with Alexa Fluor 488 anti-Rabbit IgG (no. A11008) (Invitrogen) in blocking buffer for 48 hr.

### Data Analysis

All results are expressed as the mean ± SEM, and statistical significance was determined using the two-tailed, unpaired Student's t test or one-way ANOVA with the Bonferroni *post-hoc* test.

## Author Contributions

T.I. and E.H. conceived and designed the study. T.I., T.H., K.F., Y.N., G.P., Y.O., and K.O. performed *in vivo* experiments. T.I., T.H., K.F., M.K., T.K., M.H., and Y.K. performed *in vitro* experiments. K.K., T.M., Y.I., and M.O. discussed the results, conceived some experiments, and provided critical reagents and comments. T.I. and E.H. wrote the manuscript.

## Supplemental Information

Document S1. Figures S1 and S2 and Tables S1–S4

Document S2. Article plus Supplemental Information
