# Supplementary material for: Translational Control of Sox9 RNA by mTORC1 Contributes to Skeletogenesis
Source: Stem Cell Reports. 2018 Jun 28;11(1):228–41. doi: 10.1016/j.stemcr.2018.05.020 (PMC6117477; doi:10.1016/j.stemcr.2018.05.020)
Supplement: Document S1. Figures S1 and S2 and Tables S1–S4 [file mmc1.pdf]

Stem Cell Reports, Volume *11*

Supplemental Information

Translational Control of *Sox9* RNA by mTORC1 Contributes to Skeletogenesis

Takashi Iezaki, Tetsuhiro Horie, Kazuya Fukasawa, Makoto Kitabatake, Yuka Nakamura, Gyujin Park, Yuki Onishi, Kakeru Ozaki, Takashi Kanayama, Manami Hiraiwa, Yuka Kitaguchi, Katsuyuki Kaneda, Takayuki Manabe, Yasuhito Ishigaki, Mutsuhito Ohno, and Eiichi Hinoi

Supplemental Fig. 1

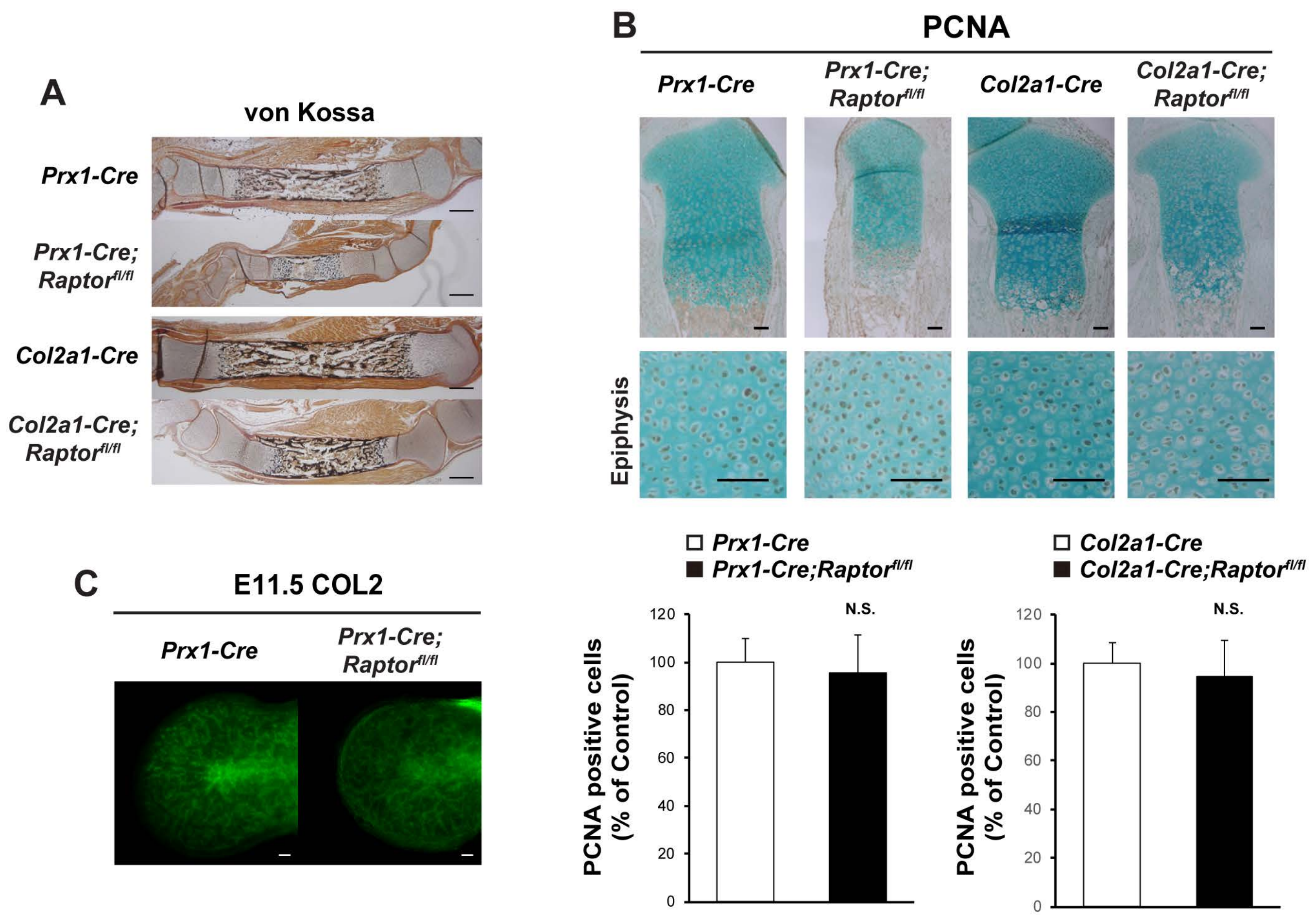

Supplemental Fig. 2

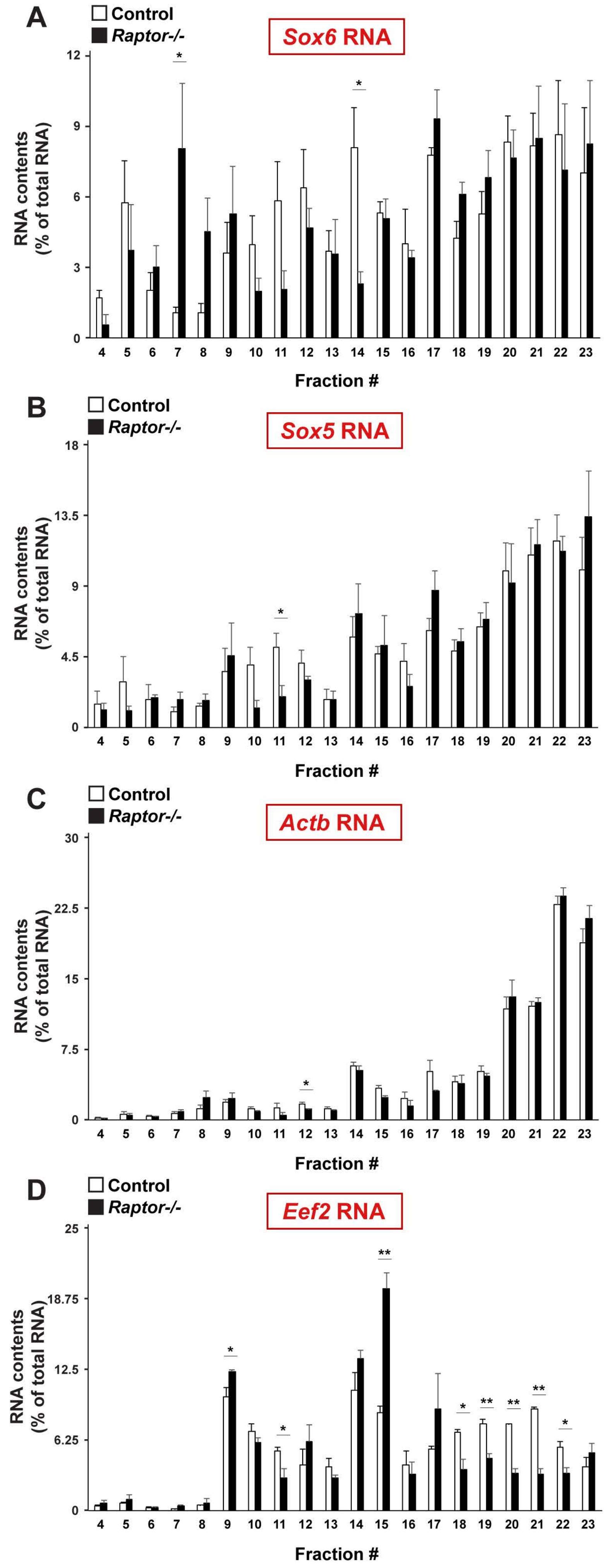

Supplemental figure legend

Supplemental Figure 1. Histological analyses, related to Figure 1 and Figure 2. (A) von Kossa stain of the tibia of *Prx1-Cre;Raptor*$^{fl/fl}$ and *Col2a1-Cre;Raptor*$^{fl/fl}$ embryos at E18.5. Bar=500 μm. (B) Immunohistochemical analyses of growth plates of *Prx1-Cre;Raptor*$^{fl/fl}$ and *Col2a1-Cre;Raptor*$^{fl/fl}$ embryos at E18.5 with anti-PCNA antibody. Bar=100 μm. N.S., not significant. (C) Whole mount immunohistochemical analyses of limb buds of *Prx1-Cre;Raptor*$^{fl/fl}$ embryos at E11.5. Bar=100 μm.

Supplemental Figure 2. Determination of RNA distribution, related to Figure 4. Polysomes from WT cells and *Raptor*-deficient cells were sedimented in sucrose density gradients to separate efficiently 40S, 60S, 80S and polysomes, followed by determination of distribution of RNA contents (n=3 independent experiments). *P <0.05, **P <0.01, significantly different from the value obtained in control cells. Statistical significance was determined using the two-tailed, unpaired Student's *t*-test.

Supplemental Table 1. List of oligonucleotides used for generation of shRNA, related to Figure 3.

Gene	Up (5'-3')	Down (5'-3')
sh4EBP1	GATCCCCGGAGGCGGTGAAGAGTCACAATTCTCGAGAATTGTGACTCTTCACCGCCTTTTTG	AATTCAAAAAGGCGGTGAAGAGTCACAATTCTCGAGAATTGTGACTCTTCACCGCCTCCGGG
sh4EBP2	GATCCCCGTCCTGGCGCCTTAATTGAAGACTTTCAAGAGAAGTCTTCAATTAAGGCGCCAGGATTTTTTGGAAAG	AATTCTTTCCAAAAAATCCTGGCGCCTTAATTGAAGACTTCTCTTGAAAGTCTTCAATTAAGGCGCCAGGACGGG

Supplemental Table 2. List of primers used for real-time PCR, related to Figure 2.

Genes	Up (5'-3')	Down (5'-3')

Runx2	CCTAGTTAGAGTGGTAGCAGAAGC	ACAGACAACGAAGAAAGTTCCCAC
Sox5	ATTGTGCAGTCCCACAGGTTG	CTGCCTTTAGTGGGCCAGTG
Sox6	CGTGCCGTCTTTGTCTATCCTGG	ACCCAAGGATGGCGTGTCTAAC
Sox9	TTTGGGTCTGCCTGGACTGTATGTG	AAGGTCTGTCCGATGTCTCTCTGC

Supplemental Table 3. List of primers used for generation of luciferase vector, related to Figure 4.

**Genes**	**Up (5'-3')**	**Down (5'-3')**
**5'UTR-Runx2-luc**	GTGAGCCAGCCCGATATTGCTTCT	ATGCTAGCCACAACAGCCACAAGTTAGC
**5'UTR-Sox6-luc**	CTCGAGAGCTGCTTTCGGCTTTC	GAAGATCTTCTTAGTTATACACGAGGATCA
**5'UTR-Sox9-luc**	CTCCGAGGAGCTCCGCTCCGACTCGCCT	CGGAGCTCCTCGGAGGGAAAACAGAGAAC
**5'UTR-Sox9^{TOPM}-luc1**	GTTTCGTTCTCTGTTTTCGGAGGGTCCTCC	AGTCGGAGCGGAGGAGGACCCTCCGAAAAC
**5'UTR-Sox9^{TOPM}-luc2**	GGAGGGAGGAGGTCCGCTCCGACTCGCCTT	TTCTCTGTTTTCGGAGGGAGGAGGTCCGCT

Supplemental Table 4. List of primers used for determination of RNA content in polysome fraction, related to Figure 4.

**Genes**	**Up (5'-3')**	**Down (5'-3')**
Actb	AAACTGGAACGGTGAAGGCGAC	CAGAAGCAATGCTGTCACCTTCC
Eef2	AGATCCGTGCCATCATGGA	TGGGCGATGACTGACATGTT
Runx2	CCTAGTTAGAGTGGTAGCAGAAGC	ACAGACAACGAAGAAAGTTCCCAC
Sox5	ATTGTGCAGTCCCACAGGTTG	CTGCCTTTAGTGGGCCAGTG
Sox6	CGTGCCGTCTTTGTCTATCCTGG	ACCCAAGGATGGCGTGTCTAAC
Sox9	TTTGGGTCTGCCTGGACTGTATGTG	AAGGTCTGTCCGATGTCTCTCTGC
